# Comparison of the cardiovascular effects of immobilization with three different drug combinations in free-ranging African lions

**DOI:** 10.1093/conphys/coac077

**Published:** 2023-01-12

**Authors:** Ashleigh Claire Donaldson, Leith Carl Rodney Meyer, Andrea Fuller, Peter Erik Buss

**Affiliations:** Department of Paraclinical Sciences, Faculty of Veterinary Science, University of Pretoria, Soutpan Road, Onderstepoort, Pretoria, Gauteng, South Africa, 0110; Centre for Veterinary Wildlife Research, Faculty of Veterinary Science, University of Pretoria, Soutpan Road, Onderstepoort, Pretoria, South Africa, 0110; Center for Zoo and Wild Animal Health, Copenhagen Zoo, Frederiksberg, Denmark, 2000; Department of Paraclinical Sciences, Faculty of Veterinary Science, University of Pretoria, Soutpan Road, Onderstepoort, Pretoria, Gauteng, South Africa, 0110; Centre for Veterinary Wildlife Research, Faculty of Veterinary Science, University of Pretoria, Soutpan Road, Onderstepoort, Pretoria, South Africa, 0110; Brain Function Research Group, School of Physiology, Faculty of Health Sciences, University of the Witwatersrand, York Road, Parktown, Johannesburg, South Africa, 2193; Department of Paraclinical Sciences, Faculty of Veterinary Science, University of Pretoria, Soutpan Road, Onderstepoort, Pretoria, Gauteng, South Africa, 0110; Centre for Veterinary Wildlife Research, Faculty of Veterinary Science, University of Pretoria, Soutpan Road, Onderstepoort, Pretoria, South Africa, 0110; Brain Function Research Group, School of Physiology, Faculty of Health Sciences, University of the Witwatersrand, York Road, Parktown, Johannesburg, South Africa, 2193; Centre for Veterinary Wildlife Research, Faculty of Veterinary Science, University of Pretoria, Soutpan Road, Onderstepoort, Pretoria, South Africa, 0110; Veterinary Wildlife Services, South African National Parks, Kruger National Park, Skukuza, South Africa, 1350; Department of Production Animal Studies, Faculty of Veterinary Science, University of Pretoria, Soutpan Road, Onderstepoort, Gauteng, South Africa, 0110

**Keywords:** skipped heart beats, medetomidine, hypertension, heart rate

## Abstract

Thirty-six free-ranging lions (12 per group) were immobilized with tiletamine–zolazepam (Zoletil 0.6 mg/kg i.m.) plus medetomidine (0.036 mg/kg i.m.) (TZM), ketamine (3.0 mg/kg i.m.) plus medetomidine (0.036 mg/kg i.m.) (KM) or ketamine (1.2 mg/kg i.m.) plus butorphanol (0.24 mg/kg i.m.) plus medetomidine (0.036 mg/kg i.m.) (KBM). During immobilization cardiovascular variables were monitored at 5-minute intervals for a period of 30 minutes. Lions immobilized with all three drug combinations were severely hypertensive. Systolic arterial pressure was higher at initial sampling in lions immobilized with KM (237.3 ± 24.8 mmHg) than in those immobilized with TZM (221.0 ± 18.1 mmHg) or KBM (226.0 ± 20.6 mmHg) and decreased to 205.8 ± 19.4, 197.7 ± 23.7 and 196.3 ± 17.7 mmHg, respectively. Heart rates were within normal ranges for healthy, awake lions and decreased throughout the immobilization regardless of drug combination used. Lions immobilized with TZM had a higher occurrence (66%) of skipped heart beats than those immobilized with KBM (25%). The three drug combinations all caused negative cardiovascular effects, which were less when KBM was used, but adverse enough to warrant further investigations to determine if these effects can be reversed or prevented when these three combinations are used to immobilize free-living lions.

## Teaser

Drug combinations used to immobilize lions work well to sedate them but have adverse effects on the way their heart functions. We compared two new drug combinations to the conventional combination and found that all caused high blood pressure, but one of the new combinations had fewer adverse effects overall.

## Introduction

Chemical immobilization of lions is an essential conservation management tool as it allows for moving individuals between isolated populations to maintain genetic diversity, collecting biological samples, attaching radio-tracking devices and treating injured individuals. It is important to be able to perform these immobilizations efficiently and safely as lions are vulnerable and important in ecosystems. African lions (*Panthera leo*) are classified as vulnerable on the IUCN Red List (IUCN, 2022) because most of their subpopulations are decreasing (Bauer *et al.,* 2015). Lions as an apex predator are essential for the health of natural ecosystems (Ripple *et al.,* 2014); they also have an aesthetic value and provide an economic contribution to the ecotourism industry (Krüger, 2005; Lindsey *et al.,* 2007).

Dissociative anaesthetics as single agents or in combination with a tranquillizer and/or sedative have been used in the immobilization of lions (Kreeger *et al.,* 2002; Fahlman *et al.,* 2005). Tiletamine combined with zolazepam (Zoletil^®^, Virbac RSA (Pty) Ltd, Halfway House, South Africa; or Telazol^®^, Zoetis, Kalamazoo, Michigan, USA) has been favoured as it has a wide safety margin and is believed to have few cardiovascular adverse effects, but major disadvantages include the lack of a reversal agent and prolonged recoveries (McKenzie, 1993; Burroughs *et al.,* 2012). Recovery time can be reduced by combining tiletamine–zolazepam (Zoletil) with medetomidine (combination TZM), a potent and highly specific α_2_-adrenoceptor agonist, and partially reversing the drug combination effects with the antagonist, atipamezole. However, the addition of medetomidine to tiletamine–zolazepam is believed to cause hypertension (Deem *et al.,* 1998; Stegmann and Jago, 2006), bradycardia (Fahlman *et al.,* 2005) and arrhythmias (Gicana *et al.,* 2021). Ketamine in combination with medetomidine (combination KM) also has been used in carnivore species (Caulkett *et al.,* 1999; Stegmann and Jago, 2006; Fahlman *et al.,* 2008; Mehmood *et al.,* 2019) including lions (Fyumagwa *et al.,* 2012). However, KM seems to result in similar cardiovascular adverse effects to TZM (Vainio and Palmu, 1989; Caulkett *et al.,* 1999).

Butorphanol tartrate, a synthetically derived opioid agonist–antagonist, has been used in combination with α_2_-adrenergic agonists, dissociative anaesthetics and tranquilizers, or other sedative drugs, to produce safer immobilization in captive and free-ranging wildlife. Drug adverse effects are reduced as the dose of each drug used in the immobilizing combination is reduced compared to combinations in which butorphanol is not included (Bush *et al.,* 2012). Butorphanol administered alone causes minimal cardiovascular effects in dogs (Girard *et al.,* 2010), but used in combination with medetomidine, it may result in bradycardia, arrhythmias and hypertension, at least in smaller felid species (Lafortune *et al.,* 2005; Blignaut, 2020). A combination of butorphanol, azaperone and medetomidine has been used previously in captive lions (Semjonov *et al.,* 2017) but caused hypertension. Ketamine, butorphanol and medetomidine (combination KBM) has been used as an immobilizing drug combination in smaller wild felids such as serval (*Leptailurus serval*) (Langan *et al.,* 2000; Moresco *et al.,* 2009; Blignaut, 2020) and bobcats (*Lynx rufus*) (Rockhill *et al.,* 2011), although significant bradycardia was observed.

Although cardiovascular effects are reported for commonly used drug combinations, many studies only report heart rate changes. Arterial blood pressure, a function of cardiac output and systemic vascular resistance, provides an improved evaluation of the cardiovascular status of an anaesthetized patient, compared to heart rate alone (Laske *et al.,* 2018; Morelli *et al.,* 2020). Monitoring blood pressure improves the outcome of anaesthesia by helping to prevent and diagnose early a wide variety of cardiovascular complications which can be caused by chemical immobilization. Immobilization of lions with TZM (Fahlman *et al.,* 2005; Jacquier *et al.,* 2006) and KM (Fyumagwa *et al.,* 2012) has been described, although only briefly, with no insight into in-depth cardiovascular effects of either drug combination. Heart rate was unaffected by immobilization with these two drug combinations in lions and was the only cardiovascular variable reported.

The aim of this study was to gain a greater understanding of the cardiovascular effects of TZM, KM and KBM in immobilized free-living African lions. We hypothesized that the synergistic effects of the different drugs in the combinations would result in differing cardiovascular effects during immobilization. To achieve this aim, intra-arterial blood pressure, heart rate and its rhythm were evaluated over a 30-minute period in lions immobilized with each drug combination.

## Materials & Methods

### Experimental procedure

All data were collected in the Kruger National Park, South Africa (24°23′52″ S, 31°46′40″ E) between April and July 2021. The study was approved by the Animal Ethics Committees of the University of Pretoria (REC 102-20) and South African National Parks (SANParks) Animal Use and Care Committee (015-20). Procedures were implemented according to the SANParks standard operating procedure for the capture, transportation and maintenance in holding facilities of wildlife. Protocols adapted from Buss and Miller (2019) were used to capture study lions. Lions were attracted to a capture site at night (between 18:00 and 04:00; average air temperature was 22.6 ± 2.7°C) with audio of hyenas feeding or a buffalo calf bellowing. A zebra carcass was used as bait to keep the lion pride occupied and in the same place for an extended period. Thirty-six free-ranging lions (23 female and 13 male) were randomly allocated to three study groups, based on the three drug combinations—tiletamine–zolazepam–medetomidine (TZM), ketamine–medetomidine (KM) or ketamine–butorphanol–medetomidine (KBM). Once a lion suitable for the study was feeding at the carcass, its body mass was estimated and a 3-ml dart (Dan-Inject International, Pietermaritzburg, South Africa) was prepared with one of the drug combinations. The dart was fired from 15 to 20 m away using a carbon dioxide pressurized dart gun (Dan-Inject International) such that the drugs were administered intramuscularly into the shoulder or upper hind leg. The intended drug dose for lions in the TZM group was 0.6-mg/kg tiletamine–zolazepam (500-mg powder formulated in the supplied diluent to 100 mg/ml, Zoletil 100, Virbac RSA (Pty) Ltd, Halfway House, South Africa) plus 0.036 mg/kg medetomidine (Metonil 40 mg/ml, Wildlife Pharmaceuticals South Africa (Pty) Ltd, White River, South Africa). The intended drug dose for lions in the KM group was 3.0-mg/kg ketamine (1-g ketamine formulated with sterile water to 200 mg/ml, Kyron Laboratories, Johannesburg, South Africa) plus 0.036-mg/kg medetomidine. The intended drug dose for lions in the KBM groups was 1.2-mg/kg ketamine plus 0.24-mg/kg butorphanol (50 mg/ml butonil, Wildlife Pharmaceuticals South Africa (Pty) Ltd) plus 0.036-mg/kg medetomidine. Once adequately immobilized (laterally recumbent and able to be safely handled), lions were blindfolded and their front limbs hobbled, transported by vehicle to a nearby (600- to 800-m away) processing site, placed on a table in left lateral recumbency and instrumented with monitoring devices. Wet bulb globe temperature was measured at the start of each immobilization using a Kestrel Heat Stress Tracker (5400, Kestrel Meters, Boothwyn Pennsylvania, USA).

A 22-gauge × 1″ intravascular catheter (Introcan, BBraun Medical Inc., Bethlehem, Pennsylvania, USA) was inserted into a dorsal pedal artery and secured in place. Intra-arterial blood pressure and heart rate were measured by using a pre-calibrated pressure transducer (Deltran II, Utah Medical, Midvale, Utah, USA) placed at the level of the heart and zeroed to the atmosphere before being connected to a PowerLab Exercise Physiology System (ML870B80, ADInstruments, Sydney, NSW, Australia) and blood pressure amplifier (ML117, ADInstruments). LabChart Software (Version 7, ADInstruments) was used to record and analyse the pressure signal generated from the PowerLab System. Systolic arterial pressure (SAP), mean arterial pressure (MAP), diastolic arterial pressure (DAP) and heart rate were recorded from 15 minutes (T_0_) after the lion became immobilized. Measurements were taken for a minute, at 5-minute intervals, over 30 minutes (T_30_). At the end of the procedure, the lion was weighed by suspending it on a stretcher from an electronic scale (Crane Scale 500kh, Miles Industrial Fasteners & Hardware CC, Benoni, South Africa) and aged according to Smuts et al. (1978) and its gender recorded. In addition, immobilized lions were branded as part of SANParks ongoing tuberculosis surveillance.

Butorphanol’s effects were antagonized (i.m.) with naltrexone (50 mg/ml, Kyron Laboratories) at twice the butorphanol dose (mg) and medetomidine’s effects were antagonized (i.m.) with atipamezole (20 mg/ml, V-Tech (Pty) Ltd, Midrand, South Africa) at 5 times the medetomidine dose (mg). All lions were monitored and protected from potential attack by other lions or hyaenas until they were fully recovered and had re-joined the pride.

### Statistical analysis

**Figure 1:**
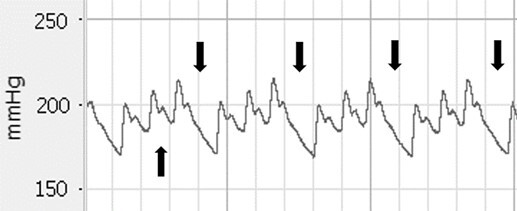
Example of the trace of mean arterial blood pressure, in the dorsal pedal artery of a lion, created by LabChart (ADInstruments). Arrows pointing downwards indicate skipped heart beats. The arrow pointing upwards indicates the dicrotic notch.

Statistical analysis was performed using RStudio version 3.6.1 (RStudio: Integrated Development for R. RStudio, PBC, Boston, MA). Data are presented as mean ± standard deviation. Heart rate, SAP, MAP and DAP were analysed offline and compiled into 1-minute average time bins using the data acquisition software program LabChart (ADInstruments). In some lions, skipped heart beats were evident in the blood pressure trace; the number of skipped heart beats and number of heart beats between skips in 1-minute average time bins was determined manually using the arterial blood pressure trace produced by LabChart (ADInstruments) (Figure 1).

Physiological data collected over time were compared between groups using a linear mixed effects model (fixed variables: time, drug combination, sex, age, body mass, body condition, wet bulb globe temperature; random variable: lion ID) with a temporal autocorrelation term. Significant values were compared using a Bonferroni correction for multiple pairwise comparisons to determine where differences occurred. One way ANOVA was used to determine if there were differences between the mean body mass of each group. A chi-square test for independence was used to compare the prevalence of skipped heart beats between lions immobilized with each drug combination, defined as the number of lions that exhibited skipped heart beats in each group. In animals that experienced skipped heart beats frequency of skipped beats was defined as the number of skipped heart beats per minute and was compared between groups using an ANOVA, as was the number of heart beats between skipped beats.

## Results

Lions immobilized with TZM received mean doses of 0.58 ± 0.04-mg/kg tiletamine–zolazepam and 0.034 ± 0.003-mg/kg medetomidine. Lions immobilized with KM received mean doses of 2.93 ± 0.42-mg/kg ketamine and 0.035 ± 0.005-mg/kg medetomidine. Lions immobilized with KBM received mean doses of 1.15 ± 0.13-mg/kg ketamine, 0.23 ± 0.03-mg/kg butorphanol and 0.034 ± 0.004-mg/kg medetomidine.

### Mean arterial blood pressure

**Figure 2:**
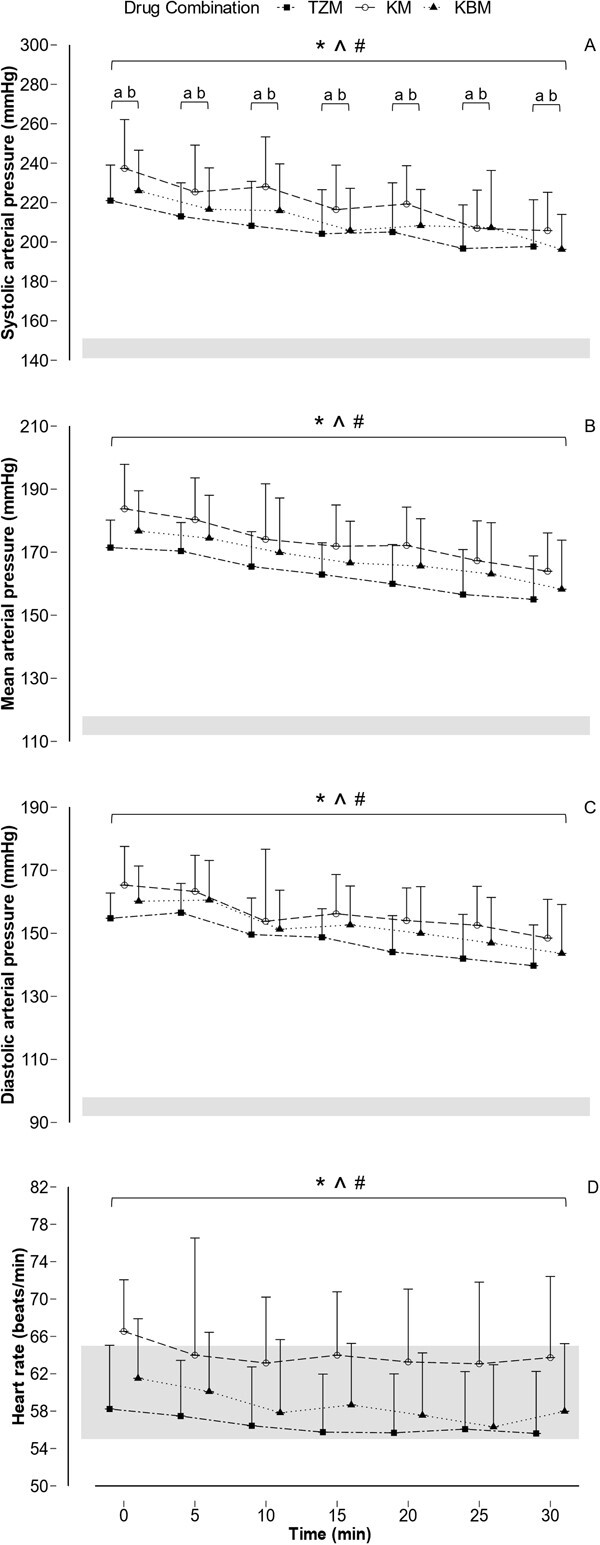
Mean and SD of (A) SAP, (B) MAP, (C) DAP and (D) heart rate in free-ranging African lions immobilized with tiletamine–zolazepam–medetomidine (TZM, *n* = 12), ketamine–medetomidine (KM, *n* = 12) or ketamine–butorphanol–medetomidine (KBM, *n* = 12). Note: Shaded areas represent expected normal range of blood pressures (White and Seymour, 2014) and heart rate in awake, unrestrained lions (Al-Naji *et al.,* 2019).^ *^P < 0.05 T_30_ vs T_0_ TZM; ^ *P* < 0.05 T_30_ vs T_0_ KM; # < 0.05 T_30_ vs T_0_ KBM; a *P* < 0.05 TZM vs KM; b *P* < 0.05 KM vs KBM.

Mean MAP at T_0_ did not differ between lions immobilized with each drug combination (TZM = 171.5 ± 8.7 mmHg; KM = 183.7 ± 14.1 mmHg; KBM = 176.7 ± 12.8 mmHg) (beta = 9.87, *t* = 1.78, *P* = 0.09) and decreased by T_30_(TZM = 155.0 ± 13.8 mmHg; KM = 164.0 ± 12.2 mmHg; KBM = 158.3 ± 15.6 mmHg) (beta = −11.47; *t* = −7.22; *P* < 0.01) (Figure 2; Supplementary Table S1). MAP did not differ between males and females (beta = 1.97; *t* = 0.23; *P* = 0.82). MAP was not affected by environmental temperature (beta = −0.13; *t* = −0.13; *P* = 0.89).

### Systolic arterial blood pressure

Mean SAP was significantly higher in lions immobilized with KM (237.3 ± 24.8 mmHg) than in those immobilized with TZM (221.0 ± 18.1 mmHg) and KBM (225.99 ± 20.6 mmHg) (beta = 15.73; *t* = 2.13; *P* = 0.04) at T_0_. Mean SAP decreased significantly to 197.7 ± 23.73-mmHg TZM, 205.8 ± 19.42-mmHg KM and 196.3 ± 17.7-mmHg KBM at T_30_ (beta = −29.29, *t* = −6.92, *P* < 0.01) (Figure 2; Supplementary Table S1). SAP did not differ between males and females (beta = −10.30; *t* = −0.76; *P* = 0.46). SAP was not affected by environmental temperature (beta = 0.35; *t* = 0.23; *P* = 0.82).

### Diastolic arterial blood pressure

Mean DAP at T_0_ did not differ between lions immobilized with each drug combination (TZM = 154.8 ± 8.0 mmHg; KM = 165.3 ± 12.3 mmHg; KBM = 160.2 ± 11.2 mmHg) (beta = 8.13; *t* = 1.56; *P* = 0.13) and decreased by T_30_ (TZM = 139.7 ± 12.9 mmHg; KM = 148.5 ± 12.3 mmHg; KBM = 143.6 ± 15.5 mmHg) (beta = −15.73; *t* = −9.31; *P* < 0.01) (Figure 2; Supplementary Table S1). DAP did not differ between males and females (beta = −0.84; *t* = −0.10; *P* = 0.92). DAP was not affected by environmental temperature (beta = −0.01; *t* = −0.01; *P* = 0.99).

### Heart rate

Heart rates at T_0_ were 58 ± 7 beats/min (TZM), 67 ± 6 beats/min (KM) and 62 ± 6 beats/min (KBM) and did not differ between drug combinations (beta = 4.37; *t* = 1.54; *P* = 0.13). Mean heart rate had decreased significantly at T_30_ to 56 ± 7 beats/min (TZM), 64 ± 9 beats/min (KM) and 58 ± 7 beats/min (KBM) (beta = −3.51; *t* = −2.73; *P* < 0.01) (Figure 2; Supplementary Table S2). Heart rate did not differ between males and females (beta = 3.55; *t* = 0.74; *P* = 0.47). Heart rate was not affected by environmental temperature (beta = 0.77; *t* = 1.48; *P* = 0.15).

### Skipped heart beats

**Figure 3:**
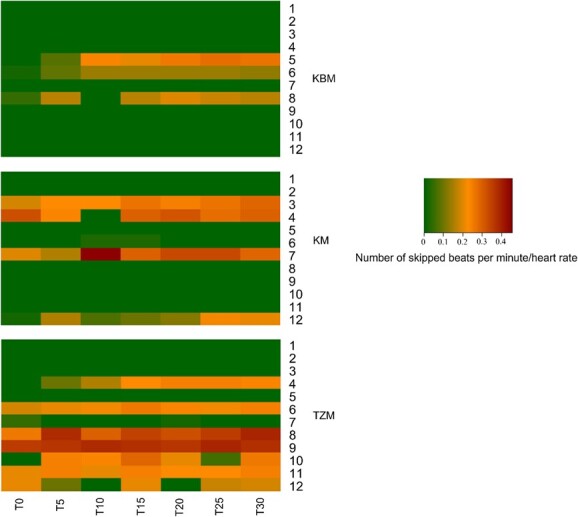
Heat map of occurrence and severity skipped heart beats (expressed as skipped beats per minute relative to heart rate) in lions immobilized with tiletamine-zolazepam-medetomidine (TZM), ketamine-medetomidine (KM) or ketamine-butorphanol-medetomidine (KBM).

Lions immobilized with TZM had a higher prevalence of skipped heart beats than those immobilized with KBM (*P* = 0.04), with 67% of lions immobilized with TZM and 25% of lions immobilized with KBM experiencing skipped heart beats (Figure 3). Prevalence of skipped heart beats between lions immobilized with TZM and KM (*P* = 0.22) and between lions immobilized with KM and KBM (*P* = 0.41) did not differ.

The frequency at which skipped heart beats occurred remained constant over time at 12 ± 5 skipped beats/minute (TZM), 3 ± 7 skipped beats/minute (KM) and 9 ± 1 skipped beats/minute (KBM) (*P* = 0.10), as did the number of normal heart beats between each skipped heart beat at 3 ± 1 beats (TZM), at 4 ± 1 beats (KM) and 4 ± 0 beats (KBM) (*P* = 0.84). In the animals where skipped heart beats occurred, there was no difference in the frequency of skipped heart beats (beta = 2.77; *t* = 0.72; *P* = 0.50) or the number of normal heart beats between skipped heart beats (beta = −0.06; *t* = −0.05; *P* = 0.97) between drug combinations.

One lion immobilized with TZM (Lion 9 in TZM group, Figure 3) exhibited intermittent double skipped heart beats, with 17% of skipped heart beats being double skips and 83% being single skipped heart beats. One lion immobilized with KM exhibited intermittent double skipped heart beats (Lion & KM group, Figure 3), with 11% of skipped heart beats being double skips and 89% being single skipped heart beats. No lions immobilized with KBM exhibited double skipped heart beats.

Body mass did not differ between lions immobilized with each drug combination at 149.6 ± 21.0 kg (TZM), 136.3 ± 28.7 kg (KM) and 164.0 ± 36.6 kg (KBM) (*F* = 2.43, *P* = 0.10). Age did not differ between lions immobilized with each drug combination at 6.2 ± 1.8 years (TZM), 5.0 ± 2.9 years (KM) and 5.5 ± 3.0 years (KBM) (*F* = 0.51, *P* = 0.61).

## Discussion

Lions immobilized with all three drug combinations exhibited hypertension throughout the monitored immobilization (T_0_ to T_30_), although blood pressure decreased significantly by between 15 and 30 mmHg over this period. SAP was highest in lions that received KM, averaging 237 mmHg at the start of monitoring. Despite blood pressures being elevated well above the normal values for an awake lion, the heart rates of lions were mostly within reported ranges for awake lions, throughout the immobilization with all the drug combinations. Nevertheless, heart rates also decreased over the period by 2 to 4 beats. Skipped heart beats were observed in 16 of the 36 lions, with a higher prevalence in lions immobilized with TZM than in those immobilized with KBM, while prevalence in lions immobilized with KM did not differ from those immobilized with TZM or KBM. There was no difference in the frequency of skipped beats or the number of normal heart beats between skipped beats between drug combinations. Intermittent double skipped beats were exhibited by one lion immobilized with TZM and one with KM.

Our study improves on previous studies due to the greater depth in monitoring the cardiovascular system. There are studies where cardiovascular measures are reported in immobilized lions (Bush *et al.,* 1978; Fahlman *et al.,* 2005; Wenger *et al.,* 2010; Reilly *et al.,* 2014; Semjonov *et al.,* 2017); however, these studies only measured heart rate, with the exception of one study that measured non-invasive blood pressure (Semjonov *et al.,* 2017). A limitation of our study is the lack of reference ranges for blood pressure in healthy, awake lions. Therefore, we compared lions’ blood pressure measurements to predicted “normal” values based on allometric scaling calculations, which are based solely on body mass (White and Seymour, 2014). Although we believe that these values are useful for comparative purposes, they require validation. Another limitation of this study was the absence of an electrocardiogram, which made it impossible to classify arrhythmias to degree level. Gender differences in blood pressure have been observed in mammals and are thought to be related to levels of androgens such as testosterone (Reckelhoff, 2001). Hormones were not measured in our study and, as such, their effects on sympathetic and parasympathetic pathways could not be quantified.

Lions immobilized with all three drug combinations were severely hypertensive. Hypertension in domestic cats is defined as SAP greater than 160 mmHg and/or diastolic pressure greater than 100 mmHg (Stepien, 2010), with systolic pressures between 160 and 179 mmHg considered moderate and greater than 180 mmHg considered severe (Taylor *et al.,* 2017). Healthy, unrestrained animals in the mass range of our study animals are predicted to have an SAP of 140–150 mmHg, MAP of 112–118 mmHg and DAP of 92–98 mmHg (White and Seymour, 2014). Mean SAP, MAP and DAP for all three treatment groups was 50, 35 and 40 mmHg higher, respectively, than allometrically calculated. Hypertension appears to be a common consequence of the drug combinations used to immobilize lions, with elevations of 20–40 mmHg recorded in lions immobilized with xylazine–ketamine (Omóbòwálé *et al.,* 2017), butorphanol–azaperone–medetomidine (Semjonov *et al.,* 2017) and ketamine–midazolam (Aguilar *et al.,* 1997). Hypertension has also been observed in other felid species immobilized with xylazine–midazolam–ketamine (Siberian tiger, *Panthera tigrisaltaica*; Curro *et al.,* 2004), medetomidine–midazolam–ketamine (Siberian tiger; Curro *et al.,* 2004), TZM (Cheetah, *Acinonyx jubatus*; Stegmann and Jago, 2006; Deem *et al.,* 1998) and medetomidine–ketamine (Cheetah & domestic cats, *Feliscatus*; Stegmann and Jago, 2006; Dobromylskyj, 1996).

The hypertension observed in our lions is attributed primarily to the actions of medetomidine. α_2_-Adrenoceptor agonists (such as medetomidine) affect cardiovascular function through the activation of both central and peripheral receptors (Sinclair, 2003). Initially, activation of peripheral receptors in the vasculature causes vasoconstriction resulting in increases in systemic vascular resistance (Haskins *et al.,* 1986; Lammintausta, 1991; Pypendop and Verstegen, 1998), with concurrent increases in systemic blood pressure. The increase in arterial blood pressure activates the arterial baroreflex, which elicits a reflex-mediated increase in cardiac vagal nerve activity, a reduction in heart rate and a subsequent decrease in cardiac output and blood pressure (McMurphy *et al.,* 2018). Activation of central receptors results in sympatholytic effects that may amplify these effects on the heart and reduce vascular tone and vascular resistance (Vongpatanasin *et al.,* 2011), further decreasing blood pressure.

Lions in this study did not have a biphasic blood pressure response that is usually seen when α_2_-agonists are used; they remained hypertensive throughout the immobilization procedure. Prolonged hypertension lasting 60 minutes has been reported in dogs given 0.03 mg/kg of medetomidine alone (Cullen and Reynoldson, 1993), and reduced blood pressures following initial hypertension are less likely when medetomidine doses of 0.03–0.05 mg/kg are used in dogs (Räihä *et al.,* 1989; Sap and Hellebrekers, 1993). The persistent hypertension in our study lions at T_30_ was already significantly lower (15–30 mmHg) than at T_0_ (Figure 2) and the time over which measurements were taken may simply not have been long enough to observe a return to normotensive values. Decreased drug effects due to redistribution and metabolism likely explain the decreasing blood pressure over time in the immobilized lions, irrespective of the drug combination used.

It is possible that the prolonged hypertension may also have been a consequence of centrally mediated sympathomimetic effects of ketamine and tiletamine. Blockade of noradrenaline reuptake by these drugs results in an increase in circulating catecholamine concentrations and their ionotropic, chronotropic and dromotropic effects on the heart (White and Ryan, 1996; Wagner and Hellyer, 2000; Koli *et al.,* 2021), which could have countered the reflex baroreceptor response that causes slowing of the heart rate, and the expected biphasic blood pressure response that normally occurs when α_2_-agonist are administered on their own (Curro *et al.,* 2004; Ebner *et al.,* 2007). Furthermore, the higher SAP observed in lions immobilized with KM compared to those immobilized with KBM in this study may be explained by the effect of higher doses of ketamine on the cardiovascular system; the ketamine dose in KM was 2.5 times that for KBM. In human patients (Christ *et al.,* 1997) and dogs (Dowdy and Kaya, 1968; Traber *et al.,* 1971), ketamine increases arterial blood pressure, and it is well known that when domestic cats are anaesthetized with KM they develop a persistent hypertension (Dobromylskyj, 1996).

The initial hypertension in the lions could also have occurred as a consequence of an excitement-induced stress response due to stimulation caused by feeding on the carcass (Ulrich-Lai and Herman, 2009). Intra-pride competition for food and fighting causes excitement that could have initially resulted in an increased sympathetic drive and higher blood pressures (Ulrich-Lai and Herman, 2009; Kasahara *et al.,* 2021). Acute stress increases sympathoadrenal activity resulting in increased secretion of catecholamines such as noradrenaline and adrenaline, and enhanced vascular tone and cardiac stimulation, causing hypertension (Zimmerman and Frohlich, 1990; Zhang and Anderson, 2014). However, catecholamines are metabolized relatively quickly (Peaston and Weinkove, 2004), so it is unlikely that this possible excitement-induced hypertension persisted throughout the immobilization.

Lions immobilized with TZM, although hypertensive, had a lower SAP than those immobilized with KM throughout the immobilization period (Figure 2). Tiletamine used as a sole agent for immobilization in cats causes increased blood pressure (Calderwood *et al.,* 1971). However, the inclusion of zolazepam, a benzodiazepine, with tiletamine is believed to counter the sympathomimetic effects of tiletamine. Benzodiazepines cause peripheral vasodilation and an associated decrease in blood pressure (Griffin *et al.,* 2013), which may explain the lower SAP in lions immobilized with TZM compared to those immobilized with KM. The difference in SAP in lions immobilized with KM compared to KBM could also in part be due to the potential vasodilatory effects of butorphanol (Trim, 1983; Greene *et al.,* 1990; Plumb, 2008). However, if these drug-induced vasodilatory effects occurred, differences in other blood pressure variables, especially diastolic pressure, would also be expected.

Despite the persistent hypertension in all lions, the heart rate of lions immobilized with all three drug combinations decreased over the 30-minute immobilization period but remained within the normal limits expected of healthy, awake lions (Figure 2). As with blood pressure, decreased drug effects resulting from redistribution and metabolism likely account for these decreasing heart rates over time. As with blood pressure, higher heart rates at T_0_ compared to T_30_ could also have occurred as a consequence of an excitement-induced stress response (Ulrich-Lai and Herman, 2009). Although the heart rate significantly decreased over time for all the drug combinations, this decrease was small, on average 2 to 4 beats/minute, and likely of little clinical relevance.

That the heart rates in the lions were mostly within a normal range for all three drug combinations was an unexpected finding because α_2_-agonists, particularly medetomidine, are known to cause bradycardia and bradyarrhythmias (Haskins *et al.,* 1986; Lammintausta, 1991; Pypendop and Verstegen, 1998), as a result of diminished sympathetic tone and the baroreceptor reflex (Sinclair, 2003). Heart rates are decreased in domestic cats following medetomidine administration (Stenberg *et al.,* 1987; Savola, 1989; Vaha-Vahe, 1990). Butorphanol, when used alone or in drug combinations, also can decrease heart rates (Trim, 1983; Verstegen and Petcho, 1993; Selmi *et al.,* 2002; Kalema-Zikusoka *et al.,* 2003; Wenger *et al.,* 2010), most likely through an increase in parasympathetic tone (Plumb, 2008). That bradycardia did not occur could be explained by the countering sympathomimetic effects of ketamine (Tweed *et al.,* 1972; Craven, 2007) and tiletamine (Hellyer *et al.,* 1988; Wilson *et al.,* 1993), a well-documented effect of dissociative anaesthetics, which results in increased heart rates (White, 1982; Wright, 1982).

Despite maintaining normal heart rates throughout the immobilization, some of the lions in each group experienced skipped heart beats, which resulted in arrhythmias. Vagal-induced arrhythmias, including first- and second-degree atrioventricular (AV) blocks, are commonly reported adverse effects of α_2_-agonists (Vainio and Palmu, 1989; Short, 1991; Sinclair, 2003; Cardoso *et al.,* 2011; Saponaro *et al.,* 2013). An AV block is a condition in which impulse conduction from the atria to the ventricles is delayed or blocked (Lev, 1964). In this study, electrocardiography was not used, and arrhythmias were diagnosed morphologically from the intra-arterial blood pressure traces; therefore, we could not classify the AV blocks to degree level. The finding of AV blocks in lions immobilized with all drug combinations, with no difference in the pattern, implies that medetomidine, the common agent in the drug combinations, was most likely responsible. It is likely that the other drugs used in the immobilizing combinations affected the frequency of occurrence of these AV blocks. Goats immobilized with tiletamine–zolazepam–xylazine had greater frequency of arrhythmias, likely caused by AV blocks, than when they were immobilized with ketamine–xylazine (Gicana *et al.,* 2021), similar to this study where lions in the KM and KBM groups had a lower occurrence of these arrhythmias than those in the TZM group. A larger sample size may have revealed statistical differences in patterns of skipped heart beats between groups as seems to be indicated by the heat map (Figure 3). Future studies should also look at whether other physiological variables affected the frequency of occurrence and severity of skipped heart beats.

The major clinical cardiovascular concern for lions immobilized with TZM, KM or KBM is hypertension. This severe acute hypertension is likely caused by vasoconstriction that may result in reduce blood flow to tissues and organs, resulting in hypoperfusion and subsequent ischaemia (Long and Kirby, 2008). Furthermore, organs that have a rich arteriolar supply, like the eyes, brain, kidneys and myocardium, are particularly vulnerable to injury caused by the mechanical damage from high pressures (Taylor *et al.,* 2017). Although conscious individuals with first- or second-degree AV blocks usually show no clinical signs (Iwasa *et al.,* 2019), the consequences of these during immobilization, especially when perfusion is already low, is also a potential clinical concern. Therefore, the consequence of the skipped heart beats observed in the immobilized lions should be determined. First-degree AV blocks are common in young, healthy cats due to high vagal tone and are mostly asymptomatic (Hildebrandt *et al.,* 2011). Second-degree AV blocks are generally left untreated in domestic cats provided that the heart rate is maintained at the level needed to pump adequate blood for normal body functioning (Hildebrandt *et al.,* 2011).

Future studies not only should determine the clinical relevance of the cardiovascular adverse effects observed in this study but also should focus on determining the precise mechanisms causing them. Such studies should also investigate drugs that could be used to prevent or reverse these adverse effects during immobilization. Certain drugs may be good candidates for this purpose, for example, the peripheral α -adrenoceptor antagonist vatinoxan. Vatinoxan has limited ability to penetrate the blood–brain barrier and, when co-administered with α_2_-adrenoceptor agonists, it does not impact on the quality of muscle relaxation and sedation but attenuates the negative cardiovascular effects caused by these agonists (Jaeger *et al.,* 2019; Einwaller *et al.,* 2020, 2022).

Conservation programmes that involve the immobilization of animals benefit from using procedures that are supported by information on the physiological responses of the animals. Animal welfare is an essential part of conservation and ensuring that procedures used to treat, research, and translocate wildlife cause as little harm as possible is crucial. This study has revealed that immobilized lions experience cardiovascular derangements and need to be closely monitored to reduce potential morbidity risks. By the time the immobilizing drugs were antagonized all lions were still severely hypertensive; it is unknown if, and for how long after recovery, the hypertension persisted. Improving immobilizing protocols will not only improve the welfare of individual lions but have direct conservation consequences for this vulnerable species.

## Conclusion

We found that TZM, KM and KBM resulted in clinically severe hypertension in immobilized lions. The drug combinations did not change heart rates such that they were different from those expected for a healthy lion at rest, but they did result in cardiac arrhythmias. The negative cardiovascular effects were less when KBM was used to immobilize free-living lions, compared to TZM and KM. KM caused more severe hypertension in lions than TZM or KBM did. Because the cardiovascular adverse effects of these important drug combinations are of clinical concern, future studies are needed to understand their consequences and mechanisms and to determine the best way to reverse or prevent them from occurring during the chemical immobilization of free-living lions.

## Funding

This study was supported by research funding from the Copenhagen Zoo.

## Supplementary material

Supplementary material is available at *Conservation Physiology* online.

## Data availability

The data underlying this article are available in the article and in its online supplementary material.

## Supplementary Material

Web_Material_coac077
