# Supplementary material for: Comparison of the cardiovascular effects of immobilization with three different drug combinations in free-ranging African lions
Source: Conserv Physiol. 2023 Jan 12;11(1):coac077. doi: 10.1093/conphys/coac077 (PMC9835075; doi:10.1093/conphys/coac077)
Supplement: Web_Material_coac077 [file web_material_coac077.zip › Supplementary Table S1.docx]

Table S1. Mean, diastolic and systolic arterial pressures in lions immobilized with Zoletil-medetomidine (TZM), ketamine-medetomidine (KM) or ketamine-butorphanol-medetomidine (KBM) over a 30-minute period (n = 12 per drug combination). (n = 12; ^°^n = 11)

| Time | MAP (mmHg) | | | | | | DAP (mmHg) | | | | | | SAP (mmHg) | | | | | |
| --- | --- | --- | --- | --- | --- | --- | --- | --- | --- | --- | --- | --- | --- | --- | --- | --- | --- | --- |
|  | Mean | SD | Mean | SD | Mean | SD | Mean | SD | Mean | SD | Mean | SD | Mean | SD | Mean | SD | Mean | SD |
|  | TZM | | KM | | KBM | | TZM | | KM | | KBM | | TZM | | KM | | KBM | |
| 0 | 171.5 | 8.7 | 183.7 | 14.1 | 176.7 | 12.8 | 154.8 | 8.0 | 165.3 | 12.3 | 160.2 | 11.2 | 221.0^a^ | 18.1 | 237.3^bc^ | 24.8 | 226.0^a^ | 20.6 |
| 5 | 170.4 | 9.0 | 180.3 | 13.3 | 174.4 | 13.6 | 156.5 | 9.3 | 163.3 | 11.5 | 160.5 | 12.6 | 213.0*^a^ | 17.0 | 225.4*^bc^ | 23.8 | 216.6*^a^ | 21.0 |
| 10 | 165.4^*^ | 11.1 | 174.1^*^ | 17.6 | 169.9^*^ | 17.3 | 149.6^*^ | 11.6 | 153.8^*^ | 22.9 | 151.3^*^ | 12.4 | 208.3*^a^ | 22.5 | 228.0*^bc^ | 25.3 | 215.9*^a^ | 23.8 |
| 15 | 162.9^*^ | 10.1 | 171.9^*^ | 13.1 | 166.6^*^ | 13.2 | 148.7^*^ | 9.1 | 156.2^*^ | 12.5 | 152.7^*^ | 12.3 | 204.2*^a^ | 22.4 | 216.5*^bc^ | 22.6 | 205.9*^a^ | 21.4 |
| 20 | 160.0^*^ | 12.5 | 172.1^*^ | 12.2 | 165.6^*^ | 15.0 | 144.1^*^ | 11.5 | 154.0^*^ | 10.4 | 150.0^*^ | 14.9 | 205.1*^a^ | 25.0 | 219.3*^bc^ | 19.4 | 208.3*^a^ | 18.4 |
| 25 | 156.6^*^ | 14.3 | 167.3^*^ | 12.6 | 163.1^*^ | 16.3 | 142.0^*^ | 14.0 | 152.6^*^ | 12.3 | 146.9^*^ | 14.5 | 196.7*^a^ | 22.2 | 207.0*^bc^ | 19.4 | 207.3*^a^ | 29.0 |
| 30 | 155.0^*^ | 13.8 | 164.0^*^ | 12.2 | 158.3^°*^ | 15.6 | 139.7^*^ | 12.9 | 148.5^*^ | 12.3 | 143.6^°*^ | 15.5 | 197.7^*a^ | 23.7 | 205.8^*bc^ | 19.4 | 196.3^°*a^ | 17.7 |

* Measurement at T_30_ significantly different from measurement at T_0_

^a^ Significantly different from animals immobilized with KM at specific sampling point

^b^ Significantly different from animals immobilized with TZM at specific sampling point

^c^ Significantly different from animals immobilized with KBM at specific sampling point
