# Supplementary material for: Comparison of the cardiovascular effects of immobilization with three different drug combinations in free-ranging African lions
Source: Conserv Physiol. 2023 Jan 12;11(1):coac077. doi: 10.1093/conphys/coac077 (PMC9835075; doi:10.1093/conphys/coac077)
Supplement: Web_Material_coac077 [file web_material_coac077.zip › Supplementary Table S2.docx]

Table S2. Heart rate of lions immobilized with Zoletil-medetomidine (TZM), ketamine-medetomidine (KM) or ketamine-butorphanol-medetomidine (KBM) over a 30-minute period (n = 12 per drug combination). (n = 12; ^°^n = 11)

| Time | Mean | SD | Mean | SD | Mean | SD |
| --- | --- | --- | --- | --- | --- | --- |
|  | TZM | | KM | | KBM | |
| 0 | 58 | 7 | 67 | 6 | 62 | 6 |
| 5 | 57 | 6 | 64 | 13 | 60 | 6 |
| 10 | 56 | 6 | 63 | 11 | 58 | 14 |
| 15 | 56 | 6 | 64 | 7 | 59 | 7 |
| 20 | 56 | 6 | 63 | 8 | 58 | 7 |
| 25 | 56 | 6 | 63 | 9 | 56 | 15 |
| 30 | 56^*^ | 7 | 64^*^ | 9 | 58^°*^ | 7 |

* Measurement at T30 significantly different from measurement at T0
